# miR-125b regulates chemotaxis and survival of bone marrow derived granulocytes *in vitro* and *in vivo*

**DOI:** 10.1371/journal.pone.0204942

**Published:** 2018-10-04

**Authors:** Chun-Wei Lee, Caroline Schoenherr, Karin Battmer, Arnold Ganser, Denise Hilfiker-Kleiner, Sascha David, Matthias Eder, Michaela Scherr

**Affiliations:** 1 Department of Hematology, Hemostasis, Oncology and Stem Cell Transplantation, Hannover Medical School, Hannover, Germany; 2 Department of Cardiology and Angiology, Hannover Medical School, Hannover, Germany; 3 Department of Nephrology and Hypertension, Hannover Medical School, Hannover, Germany; National Cancer Institute, UNITED STATES

## Abstract

The evolutionary conserved miR-125b is highly expressed in hematopoietic stem cells (HSC) enhancing self-renewal and survival. Accordingly, over-expression of miR-125b in HSC may induce myeloproliferative neoplasms and leukemia with long latency. During hematopoietic cell maturation miR-125b expression decreases, and the function of miR-125b in mature granulocytes is not yet known. We here use transplantation of miR-125b over-expressing HSC into syngeneic hosts to generate and analyse miR-125b over-expressing granulocytes. Under steady state conditions, miR-125b over-expression inhibits granulocytic chemotaxis and LPS- but not PMA- and TNFα- induced cell death. Inflammatory signals modulate the effects of miR-125b over-expression as demonstrated in a sterile peritonitis and a polymicrobial sepsis model. In particular, survival of mice with miR-125b over-expressing granulocytes is significantly reduced as compared to controls in the polymicrobial sepsis model. These data demonstrate inflammation dependent effects of miR-125b in granulocytes and may point to therapeutic intervention strategies in the future.

## Introduction

MicroRNAs (miRNAs) are small non-coding RNAs involved in post-transcriptional regulation of gene expression [1]. Functional analyses suggest involvement of miRNAs in multi-target regulation of signaling pathways and/or cellular phenotypes, such as differentiation, proliferation, and apoptosis [2–5]. However the precise physiological function of individual miRNAs in specific types of cells still remains elusive.

The evolutionary conserved miR-125b is highly expressed in hematopoietic stem cells (HSC) enhancing self-renewal and survival whereas its expression decreases in committed progenitors [6–8]. It has been shown that miR-125b over-expression in HSC induces myeloproliferative disorders and myeloid as well as lymphoid leukemia with long latency in several murine transplantation models [2, 9–13]. Furthermore, apoptosis resistance has been described upon over-expression of miR-125b in myeloid cell culture models [11, 14]. Accordingly, regulators of mitochondrial apoptosis such as BAK1, BCL2, BCLw, MCL1, PUMA, BIK and BMF have been identified as miR-125b target genes [14, 15]. However, pro-apoptotic functions of miR-125b by regulating mitochondrial respiration have also been described in monocytes suggesting that miR-125b targets may individually contribute to regulation of cell survival in a cell type specific manner [4, 16–18]. Furthermore, miR-125b is involved in macrophage polarization [18] and can interfere with myeloid differentiation by regulating CBFβ, ETS1, c-JUN and STAT3 among others [4]. These data suggest different miR-125b target genes and biological effects during differentiation in different hematopoietic cell types. While most of these data derive from studies in immortalized cell lines or immature cells the function of miR-125b in normal granulocytes is not yet known.

To analyze granulocytic miR-125b expression and function in native hematopoiesis, we generated chimeric mice by transplantation of miR-125b over-expressing lineage depleted bone marrow cells in syngeneic recipients. Upon stable engraftment miR-125b over-expressing granulocytes were analyzed *in vitro* and *in vivo* under steady-state conditions as well as in a local inflammation and a polymicrobial sepsis model. We show that over-expression of miR-125b in granulocytes may modulate their chemotaxis and survival in an inflammation-dependent manner and enhances mortality in a murine cecal ligation and puncture model (CLP).

## Materials and methods

### Animal experiments

Bone marrow (BM) transplantation was performed in female C57BL/6(J) mice after myeloablative irradiation (9 Gy). Subsequently, lentivirally transduced BM progenitor cells were transplanted, and engraftment was analyzed 8 weeks post-transplantation by collecting leukocytes from bloodstream for GFP flow cytometric analysis. For induction of inflammation, chimeras were treated intraperitoneally with thioglycollate (3%) (Sigma Aldrich) for 4 hours or LPS (17.5 mg/kg) for 24 hours prior to harvest of granulocytes. Murine primary granulocytes were enriched (> 90%) by using Percoll gradient cell separation (GE Healthcare Life Sciences). The animals were housed under standard conditions with a 12 h light/dark cycle and adequate water and food.

Cecal ligation puncture (CLP) was performed by a single operator (SD) in chimeric mice from both groups (control and miR-125b) as previously described [19]. Given the expected worsening in outcome in miR-125b overexpressing mice, the severity of sepsis was reduced to a sublethal model. Briefly, under sterile conditions with inhaled isoflurane (1–3% in medical air), a midline laparotomy was placed. Sterile Q-tips were used to deliver the cecum, which was ligated at the anti-mesenteric border and puncture with 21-gauge needle and 0.5 mm of stool was gently extruded. Abdominal contents were replaced, and a two-layered closure was performed. The operator was blinded with regard to the chimeric mice. Predefined human endpoints were used according to Table 1 and mice were monitored with regard to health and behavior every 4 hours after surgery over the course of 48 hours. Once endpoint criteria were met (i.e. Score > = 5) the animals were euthanized immediately. To assure animal welfare analgesics were given (Butorphanol 1 mg/kg s.c. post-surgery and 0.8 Metamizol mixed with 500 ml drinking water *ad libitum*).

**Table 1:** Predefined human endpoints.

Score	activity grade	general condition	behaviour
**1**	very active	shiny fur, clear eyes; body cavities clean	awake, alert, curious, species-typical movements
**2**	active	Shiny fur; clear eyes, body cavities clean	alert, species-typical movements
**3**	less active	Dull fur with defects, eyes not completely opened	alert, calm, reduced movements, reduced body hygiene
**4**	limited	Dull fur, piloerection, eyes not completely opened, body cavities scruffy	calm, reduced reaction to externals stimulation, almost no body hygiene
**5**	apathetic	Piloerection, dirty fur, eyes closed, body cavities clotted, slouched posture	selfisolation; no relevant movements

All animal studies were in accordance with the German animal protection law and with the European Communities Council Directive 86/609/EEC and 2010/63/EU for the protection of animals used for experimental purposes and were approved by the Local Institutional Animal Care and Research Advisory Committee and permitted by the local authority, the Niedersächsisches Landesamt für Verbraucherschutz und Lebensmittelsicherheit [No. 33.9-42502-04-12/0815, 33.14-42502-04-12/0759, and 33.12-42502-04-17/2499].

### Cell culture and cytokines

HEK293 cells (American Type Culture Collection, ATCC) were cultured in DMEM supplemented with 10% FCS and 1% penicillin/streptomycin. Fluorouracil (5-FU) (25 mg/kg) (Medac, Hamburg, Germany) was intravenously injected to donor C57BL/6 mice (6–8 weeks old) prior to the collection of bone marrow progenitor cells. After 4 days, bone marrow progenitor cells were collected from tibia and femur and were cultured in cytokine-supplemented RPMI 1640 (Gibco) medium with 10% FCS, and 1% PS for 24 hours before exposure to lentiviral particles. The supplemental cytokines are mIL-3 (6 ng/ml), hIL-6 (10 ng/ml), mSCF (20 ng/ml).

### Lentiviral vectors

The transgene vector (S-miR-125b2-IEW) encompassing the sequence of murine pre-miR-125b-2 was constructed as previously described [4]. SIEW empty vector served as a control. All lentiviral constructs encode GFP (green fluorescent protein) as a reporter gene. The preparation of recombinant lentiviral supernatants and lentiviral transductions were performed as described earlier [20]. Briefly, murine primary BM progenitor cells (2 x 10^5^) were transduced twice by direct contact with lentiviral particles in 48-well-plates. After 24 hours, cells were washed with PBS and were again cultured in RPMI medium supplemented with cytokines for additional 24 hours. Transduction efficacy was evaluated on the date of transplantation by flow cytometry.

### Antibody and flow cytometry

PE-conjugated Gr-1 (Ly6G) antibody used for fluorescence-activated cell-sorting (FACS) to separate granulocyte subsets was purchased from Biolegend (San Diego, CA). Flow cytometric analysis was performed on a BD FACSCalibur.

### RNA isolation and quantitative real-time PCR

Trizol reagent (Invitrogen) was used to extract RNA. Total RNA was reverse-transcribed and subjected to Taqman miRNA Assay (Applied Biosystems, Foster City USA) following the manufacturer’s protocol. Sequences of miRNA-specific primers and probes (U6 served as an internal control) are identical as described before [4]. Data are shown as fold-change according the 2^ΔΔCT^ method. Reactions were performed using an ABI7500 cycler (Applied Biosystems, Foster City, USA). Fold changes of gene expression are displayed as results.

### Cell lysis and western blotting

Cell lysis and Western blotting were carried out as previously described [4]. Primary antibodies against BAK-1 and GAPDH were purchased from Cell Signaling Technology (Danvers, MA). Horseradish peroxidase-conjugated secondary antibody was obtained from Roche. Protein bands were visualized using the ChemiLucent ECL Detection System (Millipore, Billerica, MA) and were imaged by exposure to x-ray film. Densitometric analysis was performed by using VersaDoc 3000 Imaging system (Bio-Rad) and 1-D analysis Quantity One software (Bio-Rad) to quantify the relative intensities of protein bands.

### Transfilter migration assay and survival analysis

Migration assays were performed in 6.5 mm Transwell plates with 0.4 μm pore polyester membrane inserts (Corning, NY). Granulocytes (5 x 10^4^) in RPMI 1640 supplemented with 3% bovine serum albumin (BSA) were loaded into the membrane insert. Adequate amount of medium with or without C5a chemotactic agent (25 ng/ml) was placed into the lower chamber. After 5 h of incubation, cells migrated were subsequently collected for FACS analysis. For the survival analysis, granulocytes (5 x 10^5^) were stimulated with PMA (10 ng/ml), TNF-α (50 ng/ml) or LPS (1 μg/ml). After 18 hours, frequency of Annexin V+ cells was analyzed by using FACS analysis.

### ROS detection

Manganese chloride (MnCl_2_) served as a superoxide dismutase mimetic to induce ROS production as previously described [21]. ROS production was measured by using CellROX oxidative stress detection system (Invitrogen, Carlsbad USA) according to the manufacturer’s instruction. Granulocytes (5 x 10^5^) in RPMI 1640 with 3% BSA were treated with MnCl_2_ for 30 minutes in the presence of CellROX oxidative stress reagents (Deep Red). Fluorescence signals were analyzed by flow cytometry.

### Statistical analysis

Data presented in this study are noted as mean ± s.e.m. Mann-Whitney test was used to obtain *P* values. *P* values < 0.05 were considered statistically significant.

Log-rank-test was performed for statistical analysis of mice survival data. All error bars represent SD unless otherwise indicated.

## Results and discussion

### Generation and characterization of mice with miR-125b over-expressing granulocytes

**Fig 1:**
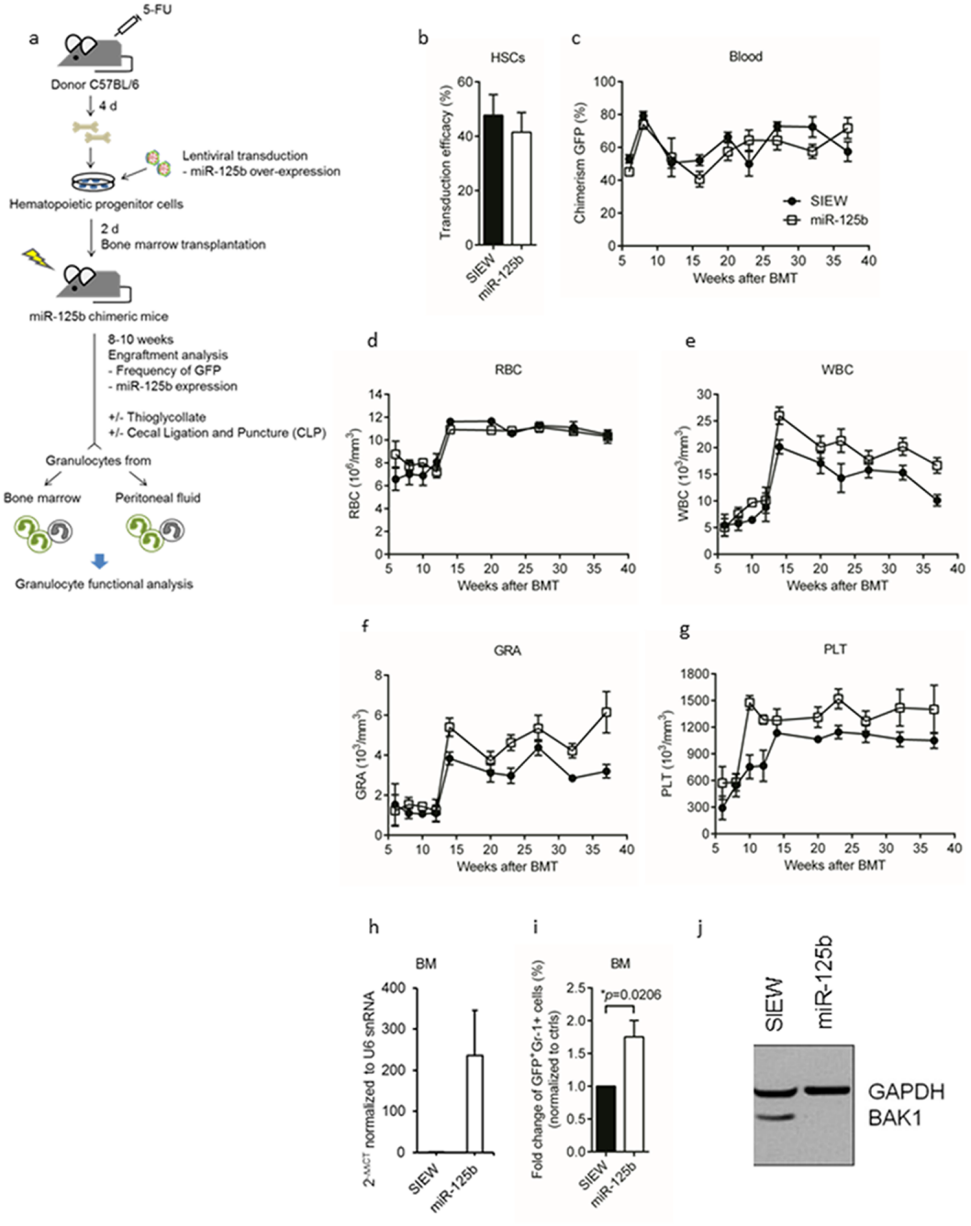
Generation of miR-125b over-expressing granulocytes from bone marrow chimeras. (a) Scheme of experimental setup. Donor mice were pre-treated with Fluorouracil (5-FU) for 4 days. Bone marrow cells were lentivirally transduced (SIEW and SIEW-miR-125b) and transplanted into lethally irradiated recipients. 8 to 10 weeks after transplantation engraftment and mir-125b expression were analyzed and granulocytes were harvested under steady state conditions after thioglycollate injection or CLP. (b) Transduction efficacy of BM cells after lentiviral transduction was analyzed by FACS analysis of GFP^+^ cells. Representative data are shown as mean ± s.e.m. from four independent experiments. (c) Engraftment kinetics from peripheral blood was monitored. Data represent mean ± s.e.m. from six independent experiments (n = 12 each group). (d-g) Kinetics of blood cells in chimeric mice after BMT. Representative data show results in (d) for red blood cells (RBC), (e) white blood cells (WBC), (f) platelets (PLT) and (g) granulocytes (GRA) from six independent experiments. (h) Under steady state conditions transduced granulocytes (GFP^+^ Gr-1^+^) were collected from bone marrow to analyze miR-125b expression levels and (i) their frequency. Expression of miRNA was normalized to U6 snRNA and relative expression levels are shown as 2^-ΔΔCT^ compared to control cells. Data represent mean ± s.e.m of four independent experiments. (j) BAK-1 protein expression in miR-125b over-expressing BM cells. BM cells were sorted for GFP^+^ populations by using flow cytometry. The protein level was analyzed by Western blotting. Data is displayed as one of two independent experiments.

To generate miR-125b over-expressing granulocytes lineage-depleted bone marrow cells were lentivirally transduced with control (SIEW, transduction efficacy: 47.7%) and or miR-125b transgenes (miR-125b, transduction efficacy: 41.5%) before transplantation into lethally irradiated syngeneic recipients (Fig 1a and 1b). Stable engraftment of GFP+ cells in peripheral blood and red blood counts were similar between control- and miR-125b mice (Fig 1c and 1d). In contrast, white blood and absolute neutrophil counts as well as platelet numbers were permanently elevated in miR-125b- as compared to control- mice starting at about 3 and 2 months after transplantation (Fig 1e–1g). Overall miR-125b was over-expressed up to 235-fold in bone marrow derived (BM-) granulocytes of miR-125b mice as compared to controls (Fig 1h). In contrast to transgenic miR-125b levels endogenous expression of miR-19b, miR-106b, miR-142-3p and miR-223 remained unchanged with lower than 1.5-fold changes between control and miR-125b transduced granulocytes (miR-19b -1.3-fold, miR-106b -1.3-fold, miR-142-3p -1.1-fold and miR-223 1.3-fold, respectively). In addition, the number of bone marrow GFP+ granulocytes was 1.8-fold higher in miR-125b- mice than in control- mice (Fig 1i). Finally, protein expression of BAK-1, a known miR-125b target [14], was reduced in FACS sorted GFP+ bone marrow cells from miR-125b- as compared to control- mice (Fig 1j).

### Function of miR-125b over-expressing granulocytes *in vitro*

**Fig 2:**
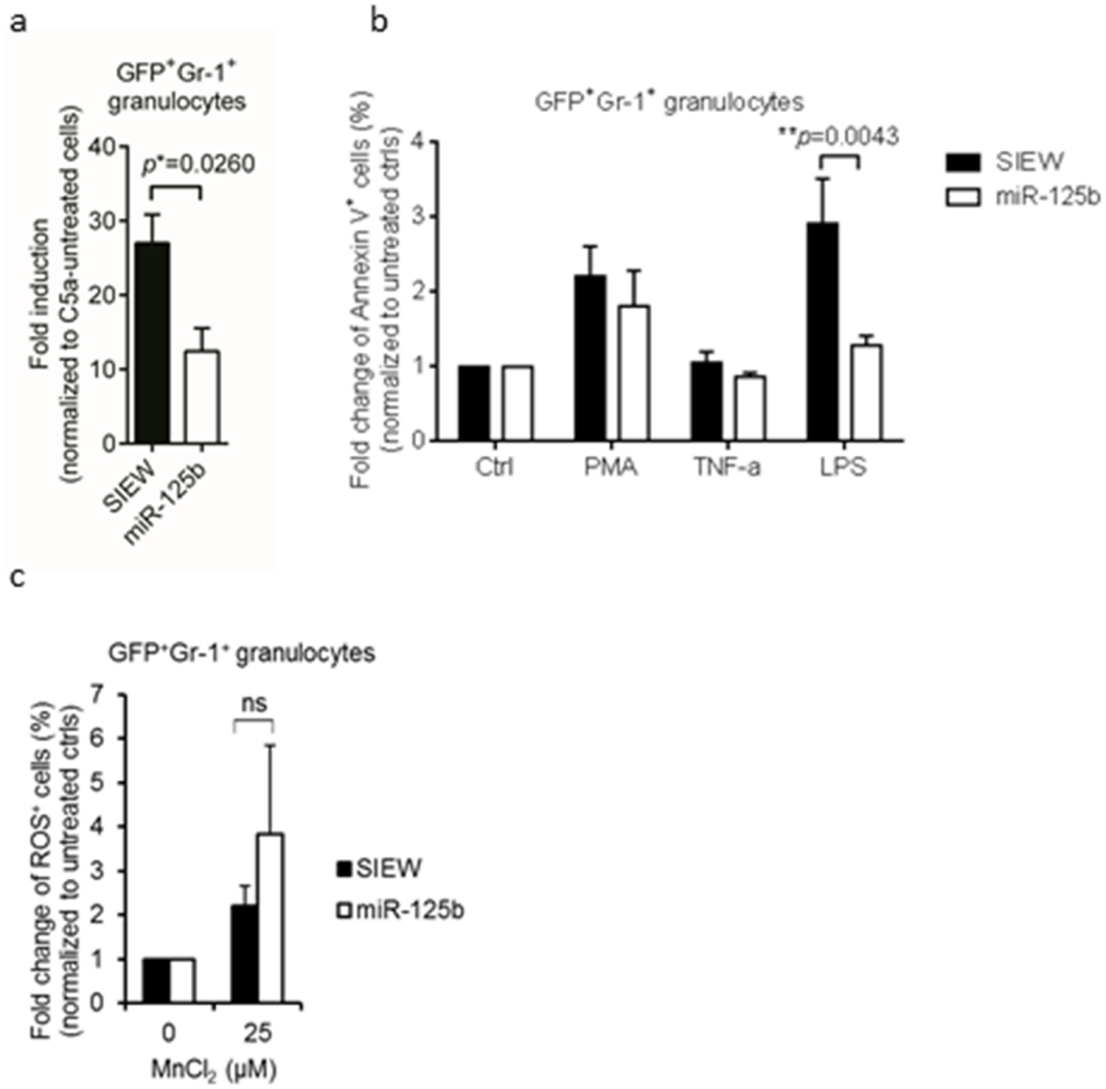
Analysis of miR-125b function in granulocytes under steady phase. (a) Chemotaxis of BM-derived granulocytes. Total bone marrow cells were harvested from BM chimeras 8 to 10 weeks post-transplantation. Granulocytes (> 90%) were enriched by using Percoll gradient cell separation. Chemotaxis of granulocytes was measured by using transfilter migration assays. Cell migration (5 x 10^4^) was measured in the absence and presence of chemotactic agent, C5a (25 ng/ml). Cells migrated to the lower chamber were collected after 5 hours of incubation for FACS analysis. Data is displayed as fold induction calculated by dividing the number of migrating cells in the treated groups by the number of migrating cells in the untreated groups. Bar diagrams represent mean ± s.e.m. n = 8 for miR-125b over-expressing granulocytes (n = 8) and for control granulocytes (n = 5) and results were pooled from five independent experiments. (b) Survival of BM-derived granulocytes. Percoll-purified granulocytes (5 x 10^5^) from BM chimeras were stimulated with PMA (10 ng/ml), TNF-α (50 ng/ml) or LPS (1 μg/ml) for 18 hours. Cell viability was determined by microscope cell counting using Trypan blue. Bar diagrams represent mean ± s.e.m. of absolute viable cell number. Data are pooled from three independent experiments. *P< 0.05, unpaired Student’s *t*-test (two-tailed). (c) Reactive oxygen species (ROS) production in BM-derived granulocytes. Granulocytes harvested from BM chimeras were treated with manganese chloride (MnCl_2_) for 30 minutes. ROS production was detected by using CellROX oxidative stress reagents (Deep Red) and granulocyte fluorescence was measured by flow cytometry. Data (mean ± s.e.m.) is presented as frequency of ROS-positive cells. Results are pooled from three independent experiments (n = 4). *P< 0.05, unpaired Student’s *t*-test (two-tailed).

Granulocytes were isolated from the bone marrow of control- and miR-125b- mice 8 weeks after transplantation (purity: >90%) when numbers of circulating granulocytes were still identical between miR-125b- and control- mice (Fig 1f). Granulocytic chemotaxis analyzed by a trans-well migration assay using C5a as a chemoattractant was significantly lower in miR-125b over-expressing granulocytes (12.5-fold induction) compared to control granulocytes (27-fold induction) (Fig 2a). Furthermore, granulocytic cell survival was enhanced in the presence of LPS but not PMA nor TNFα(Fig 2b). To analyze the impact of miR-125b on granulocytic reactive oxygen species (ROS) production granulocytes were exposed to MnCl_2_ [21]. As shown in Fig 2c, no difference in MnCl_2_-induced ROS production was observed between miR125b and control granulocytes.

### Function of miR-125b over-expressing granulocytes in localized inflammation

**Fig 3:**
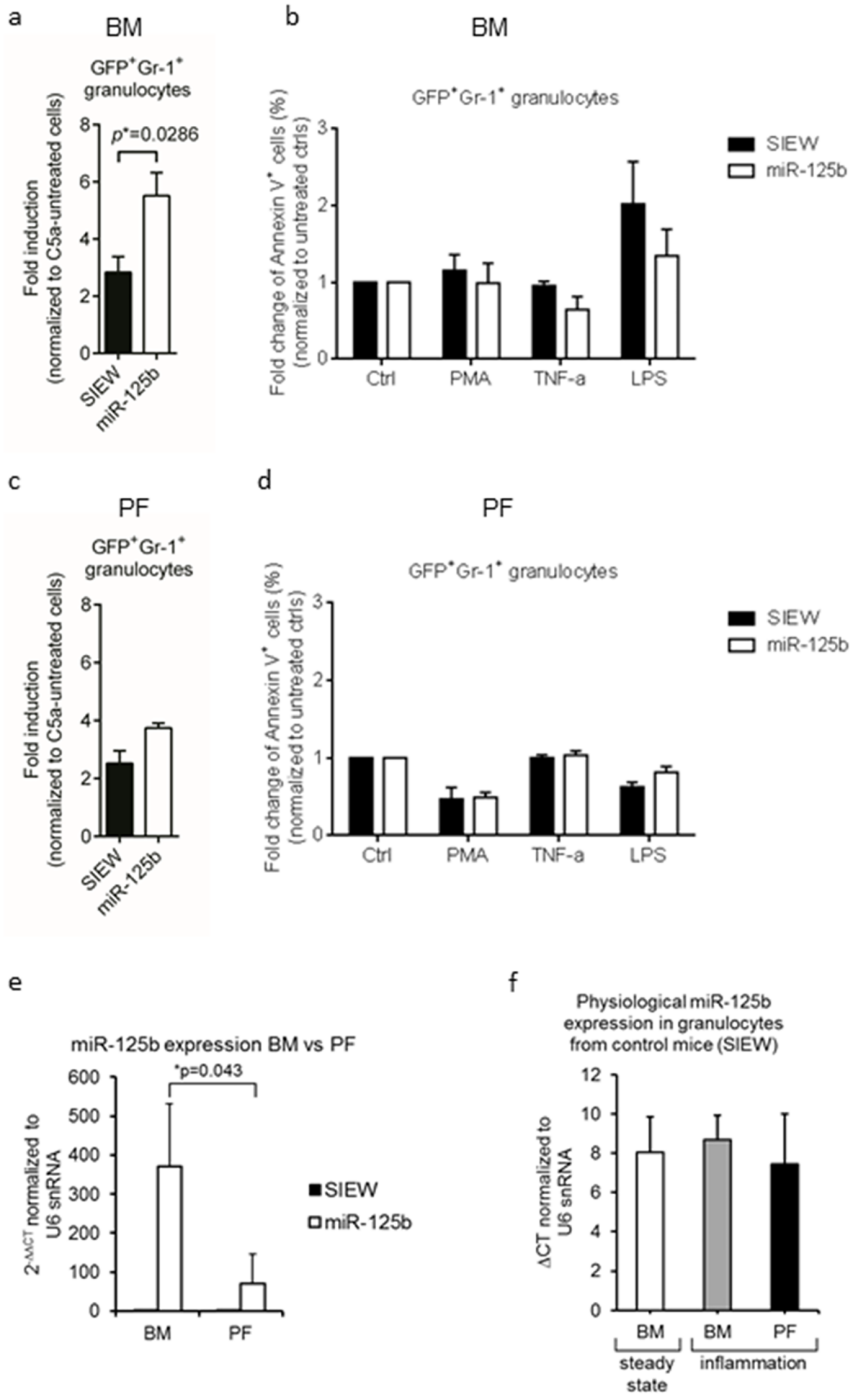
Phenotypic analyses of miR-125b expressing granulocytes after thioglycollate injection. (a,c) BM chimeras were intraperitoneally injected with thioglycollate (3%) 4 hours prior the harvest of granulocytes from (a) bone marrow and (c) peritoneal cavity. Granulocytes (5 x 10^4^) were tested in transfilter migration assays and C5a (25 ng/ml) was used as a chemotactic agent. Cells migrated to the lower chamber were collected after 5 hours of incubation for FACS analysis. Data is displayed as fold induction (calculation as described in Fig 2a). Bar diagrams represent mean ± s.e.m., n = 4 for both control and miR-125 over-expressing granulocytes, with results pooled from four independent experiments. *P< 0.05, unpaired Student’s *t*-test (two-tailed). (b,d) Granulocytes (5 x 10^5^) collected from bone marrow (b) or peritoneal cavity (d) were stimulated with PMA (10 ng/ml), TNF-α (50 ng/ml) or LPS (1 μg/ml) for 4 hours. Cell viability was determined by microscope cell counting using Trypan blue. Bar diagrams represent mean ± s.e.m. of absolute viable cell number. Data is displayed as a summary of four independent experiments. *P< 0.05, unpaired Student’s *t*-test (two-tailed). (e) miR-125b expression in PF as compared to BM derived miR-125b granulocytes determined by miR-qRT-PCR. Expression of miR-125b was normalized to U6 snRNA and relative expression levels are shown as 2^-ΔΔCT^ compared to control granulocytes (SIEW). (f) Endogenous miR-125b expression in steady-state and after thioglycollate challenge was analyzed in BM and PF-derived granulocytes from control mice (SIEW) by quantitative miR-qRT-PCR. Data from three independent experiments are presented as ΔCT values of miR-125b normalized to U6 snRNA as an internal control.

To study the role of miR-125b over-expressing granulocytes in the context of a localized sterile inflammation, miR-125b- and control- mice were treated with intraperitoneal thioglycollate as described by Ajuebor and colleagues [22]. Four hours after injection mice were sacrificed and granulocytes from bone marrow (BM) and from the peritoneal fluid (PF) were analyzed separately. In migration assays C5a-induced chemotaxis was higher in miR-125b overexpressing BM-derived granulocytes than in controls (Fig 3a) but was about 10- (control) and 2.5-fold (mir-125b) less than in granulocytes under steady state conditions (i.e. w/o thioglycollate) as shown above in Fig 2a. In addition no difference in cell survival in response to PMA, TNF-α, and LPS was observed in granulocytes from both types of mice after thioglycollate challenge (Fig 3b). C5a induced chemotaxis was similar for both peritoneal fluid (PF) and BM derived control granulocytes (black columns in Fig 3a and 3c). In contrast, chemotaxis of miR-125b PF granulocytes was reduced as compared to miR-125b BM granulocytes after thioglycollate challenge. Similar to BM-derived granulocytes we did not observe different effects of PMA, TNF-α, and LPS on cell survival of control and miR-125b PF granulocytes *in vitro* (Fig 3d). Finally, miR-125b over-expression was significantly lower in PF as compared to BM derived miR-125b granulocytes (Fig 3e). When endogenous miR-125b expression in control mice was analyzed under steady-state conditions and after thioglycollate challenge we did not observe any changes in miR-125b expression (Fig 3f). These data suggest that mostly granulocytes with lower miR-125b expression were mobilized to the site of inflammation since thioglycollate challenge does not induce changes in endogenous miR-125 expression in granulocytes.

### Effects of miR-125b over-expression in a polymicrobial sepsis model

**Fig 4:**
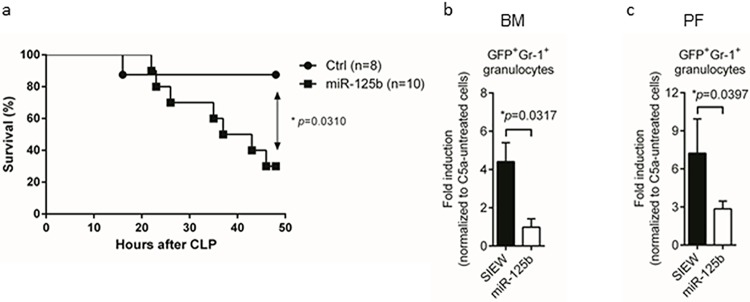
Analysis of miR-125b function in granulocytes in a sepsis model. (a) Cecal ligation puncture (CLP) was performed to induce polymicrobial sepsis in chimeric mice and human endpoints / survival were monitored every 4 hours over the course of 48 hours. Kaplan Meier survival curves represent a summary of results from 8 wildtype (1 animal euthanized / human endpoint) to 10 miR-125b transgenics (2 dead, 6 euthanized) mice from two independent experiments. *P< 0.05. (b) Granulocytes from bone marrow (BM) and (c) peritoneal fluid (PF) were analyzed 24 hours after intraperitoneal LPS injection (17.5 mg/kg) by FACS analysis of Gr-1^+^/GFP^+^ cells. Chemotaxis was analyzed as described in Fig 3. Bar diagrams represent mean ± s.e.m., from 5 control and miR-125b mice each from three independent experiments. *P< 0.05.

To characterize functional effects of miR-125b over-expression in a systemic polymicrobial sepsis model, miR-125b and control transduced mice were analyzed by cecal ligation and puncture (CLP), and survival was analyzed over 48 hours by Kaplan-Meier statistics. As shown in Fig 4a, survival of miR-125b mice was significantly reduced as compared to controls (p = 0.031). Furthermore, C5a induced chemotaxis was strongly diminished in both BM (p = 0.032) and PF (p = 0.040) derived GFP+ Gr-1+ granulocytes from miR-125b as compared to control mice (Fig 4b and 4c).

## Discussion

Here we describe a murine syngeneic transplantation model to analyze miR-125b function in mature granulocytes under both steady-state conditions and inflammatory stress. Endogenous miR-125b is expressed at low abundancy in granulocytes from control mice (Ct-value 31.6 ± 2.5) but induced 235-fold in GFP+ granulocytes from miR-125b over-expressing mice. In accordance to earlier reports, over-expression of miR-125b induces a myeloproliferative phenotype with increased numbers of leucocytes, granulocytes and platelets in our model [2, 11, 12]. However, we did not observe progression into leukemia or lethal myeloproliferation in any mouse up to twelve months after transplantation. Over-expression of miR-125b reduces C5a-induced granulocytic chemotaxis *in vitro* under steady state conditions. Furthermore, the C5a capacity to induce granulocytic chemotaxis *in vitro* is reduced in inflammation induced by both thioglycollate (Fig 3a and 3c) and polymicrobial sepsis (Fig 4b and 4c) as compared to steady-state conditions (Fig 2a). Thisreduction is stronger in control as compared to miR-125b granulocytes andand is less pronounced in miR-125b granulocytes in the thioglycollate than in the sepsis model. These data demonstrate that inflammatory signals *in vivo* differentially modulate granulocytic chemotaxis induced by C5a *in vitro* depending on miR-125b expression. Upon thioglycollate injection, mostly miR-125b low expressing granulocytes migrate into the peritoneal fluid whereas high expressing cells are found in the bone marrow (Fig 3e). Endogenous granulocytic miR-125b expression itself is not regulated at least in thioglcollate induced inflammation since it was unchanged both in granulocytes under steady-state and inflammatory conditions as well as in BM and PF- derived granulocytes in inflammation.

The molecular mechanisms how miR-125b over-expression differentially modulates chemotaxis depending on inflammatory stimuli are currently not completely understood. They may involve TLR4 signaling since miR-125b specifically inhibits granulocytic apoptosis induced by LPS but not by PMA and TNF-α under steady-state conditions (Fig 2b). Interestingly, this effect is lost upon thioglycollate injection. The essential role of miR-125b for TLR4 signaling upon LPS binding has recently been described in monocytes [18]. This may also explain the almost complete inhibition of granulocytic chemotaxis *in vitro* in the sepsis model (Fig 4b and 4c) as well as the enhanced survival of granulocytes upon LPS stimulation as discussed above. Although it is currently not known whether this link between miR-125b and TLR4 is identical in monocytes and granulocytes the data on specific LPS effects in miR-125b over-expressing granulocytes are in line with such a model. Since it is, however, difficult to specifically analyze the function of individual miR-125b targets in granulocytes by gain- or loss-of function studies overexpression of miR-125b in hematopoietic stem and progenitor cells from TLR4-/- mice and transplantation into TLR4-/- and TLR4 +/+ or +/- mice will allow to precisely address this question. The merit of the approach in our study is the analysis of granulocytic miR-125b function in a physiological model at the cost of limitations regarding identification of granulocytic miR-125 targets and their function. Finally, granulocytic death/survival is regulated by several mechanisms including mitochondrial apoptosis, ROS-production, activity of proteases from granulocytic granules and NF-ĸB among others (reviewed in [23]). Interestingly, miR-125b targets have been described in all of these contexts in different types of cells and in particular inhibition of LPS-dependent NF-ĸB signaling [9, 24–28]. Therefore, we cannot precisely decide on contributing signaling cascades beside TLR4 to the effects of miR-125b over-expression in granulocytes.

Given the observed negative effects of miR-125b on granulocytic chemotaxis *in vitro* we were hypothesizing that these mice will have a disadvantage in experimental *in vivo* model of systemic inflammation. We therefore adjusted the experimental CLP model to induce a less severe (i.e. sublethal) phenotype. Although, septic patients do not die from their infections *per se* but rather from their own pathological host response it is still obvious that without an effective immune response to an (at least initially) local infection, the organism would generally fail to fight states of infection. Our hypotheses are very well in line with the observation that miR-125b over-expressing granulocytes isolated from the peritoneal fluid in the thiogycollate model expressed miR-125b at lower levels than those remaining in the bone marrow (Fig 3e). Second, overall survival in the polymicrobial sepsis model was over 50% lower in miR-125b over-expressing mice compared to controls (Fig 4a). These data indicate that miR-125b over-expressing granulocytes might not be able to clear the local infection leading to ongoing severe systemic inflammation and ultimately death. However, we cannot determine the role of other cell types such as monocytes or macrophages derived from miR-125b overexpressing HSC and their impact on the observed phenotypes in our inflammation models.

In summary over-expression of miR-125b can negatively regulate granulocytic function in an inflammation dependent manner. This may hint to potential therapeutic intervention strategies in pathological states of altered granulocytic function in the future.

## Supporting information

S1 Fig Fig 1j.jpg. (TIF)

S1 File NC3Rs ARRIVE Guidelines Checklist 2014_Lee et al.docx. (DOCX)
