# Supplementary figures and images for: miR-125b regulates chemotaxis and survival of bone marrow derived granulocytes in vitro and in vivo
Source: PLoS One. 2018 Oct 4;13(10):e0204942. doi: 10.1371/journal.pone.0204942 (PMC6171867; doi:10.1371/journal.pone.0204942)

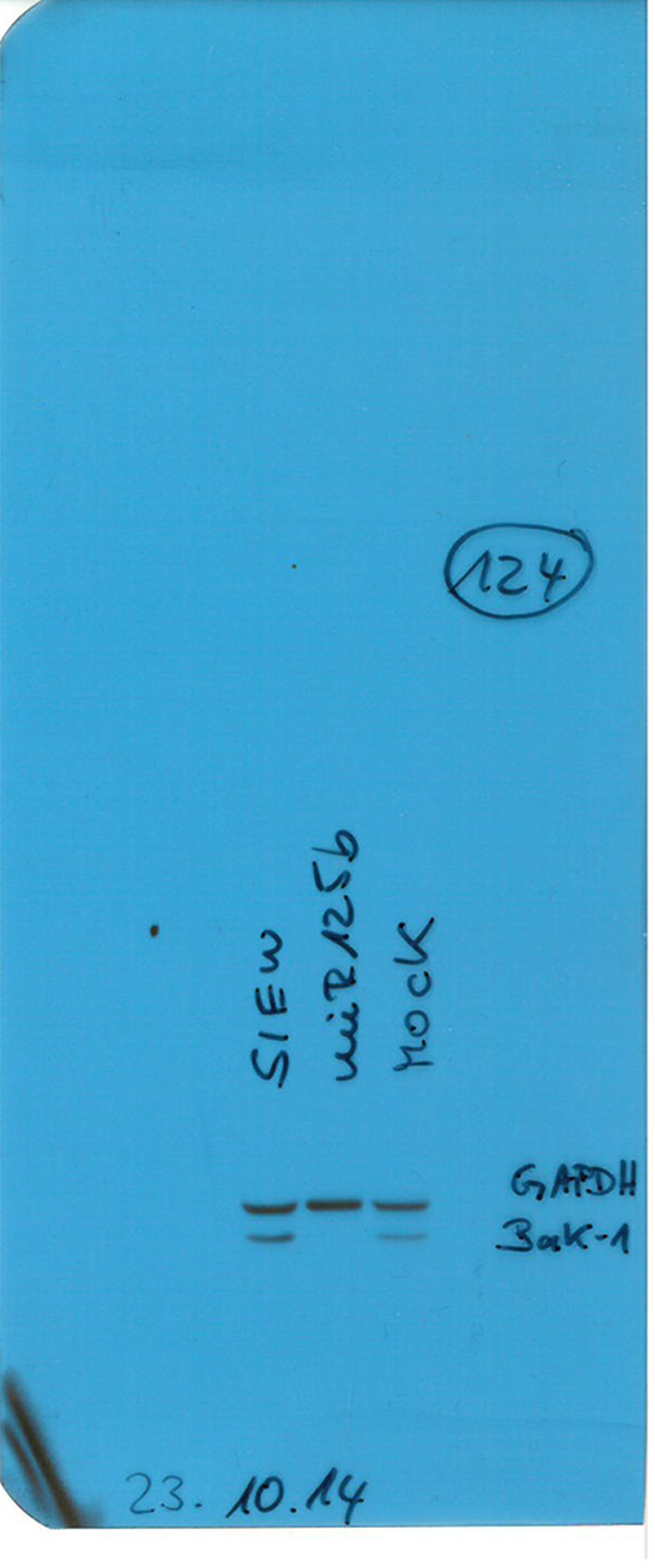

Supplement: S1 Fig — (TIF) [file pone.0204942.s001.tif]
