# Supplementary material for: miR-125b regulates chemotaxis and survival of bone marrow derived granulocytes in vitro and in vivo
Source: PLoS One. 2018 Oct 4;13(10):e0204942. doi: 10.1371/journal.pone.0204942 (PMC6171867; doi:10.1371/journal.pone.0204942)

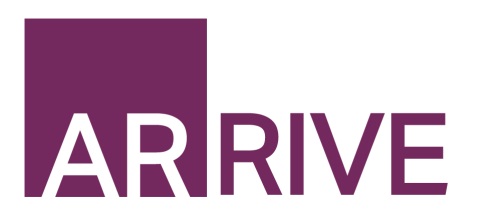


The ARRIVE Guidelines Checklist

Animal Research: Reporting In Vivo Experiments

Carol Kilkenny^1^, William J Browne^2^, Innes C Cuthill^3^, Michael Emerson^4^ and Douglas G Altman^5^

*^1^The National Centre for the Replacement, Refinement and Reduction of Animals in Research, London, UK, ^2^School of Veterinary Science, University of Bristol, Bristol, UK, ^3^School of Biological Sciences, University of Bristol, Bristol, UK, ^4^National Heart and Lung Institute, Imperial College London, UK, ^5^Centre for Statistics in Medicine, University of Oxford, Oxford, UK.*

|  | | ITEM | RECOMMENDATION | Section/ Paragraph |
| --- | --- | --- | --- | --- |
| 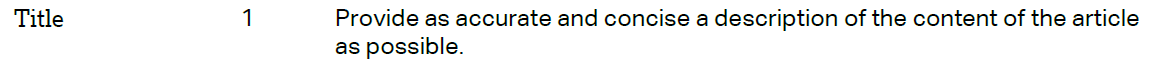 | | | title |  |
| 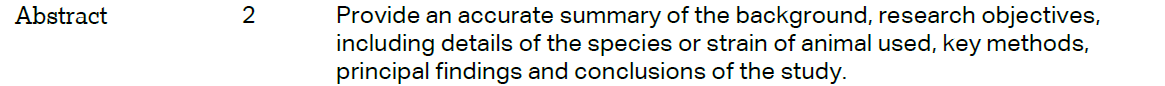 | | | abstract |  |
| INTRODUCTION | | |  |  |
| 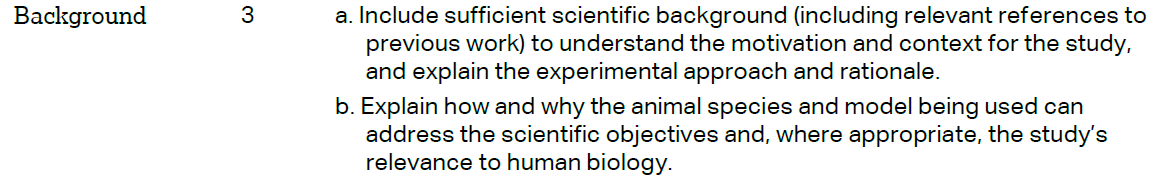 | | | Introduction paragraphs 1-3 |  |
| 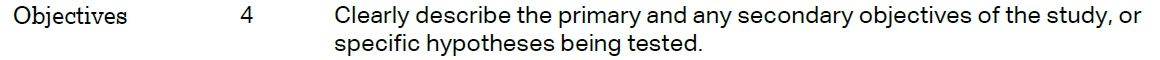 | | | paragraph 3 |  |
| METHODS | | |  |  |
| 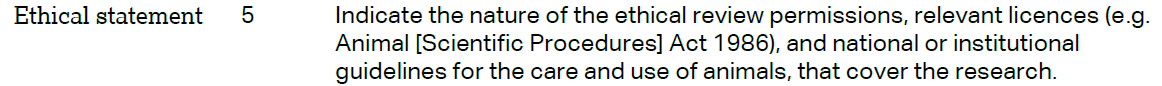 | | | M&M section, “animal experiment”s,paragraph 2 |  |
| 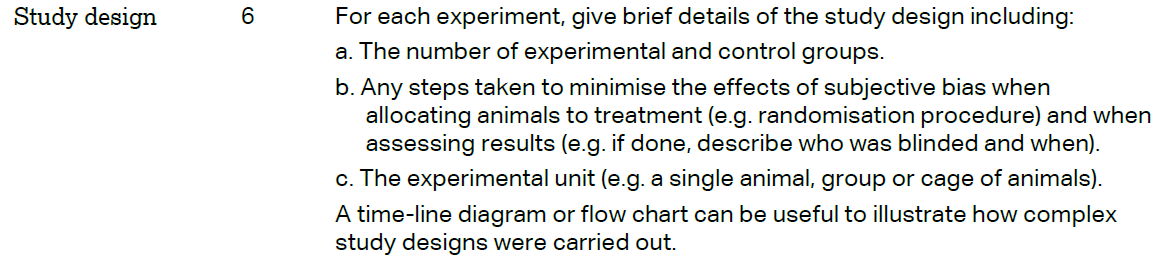 | | | M&M section, “animal exp.”paragraph 1-2 and “statistics” and figure legend 4 |  |
| 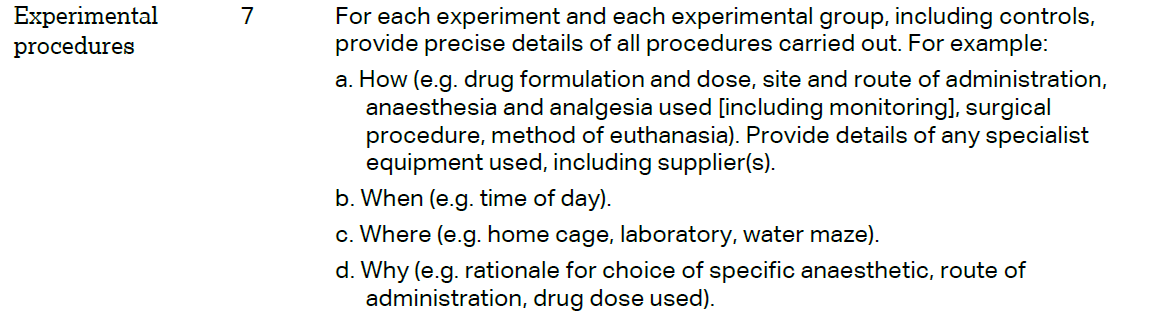 | | | M&M “animal exp.” section paragraph 1 |  |
| 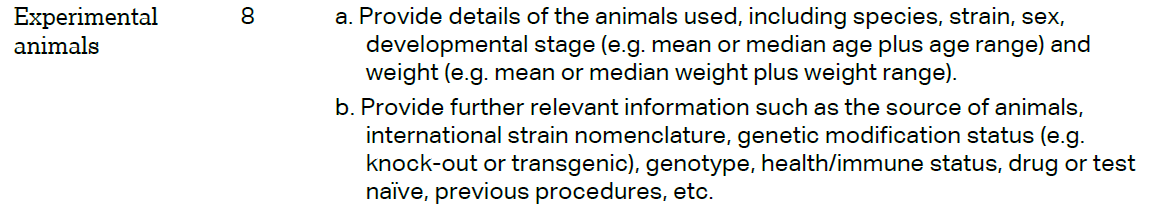 | | | M&M “animal exp.”section paragraph 1 |  |

The ARRIVE guidelines. Originally published in *PLoS Biology*, June 2010^1^

| 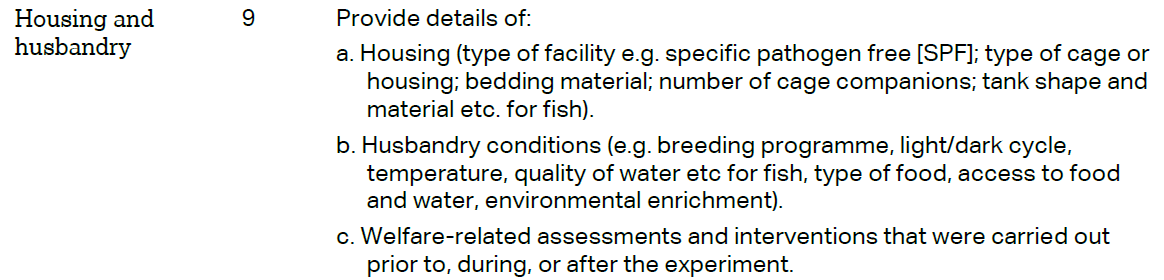 | M&M section, “animal exp” paragraph 1 | |
| --- | --- | --- |
| 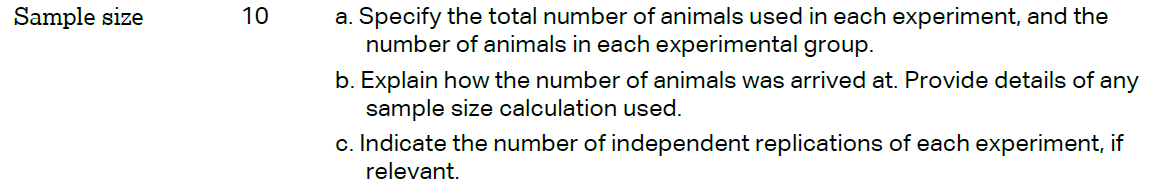 | Figure legend 4 | |
| 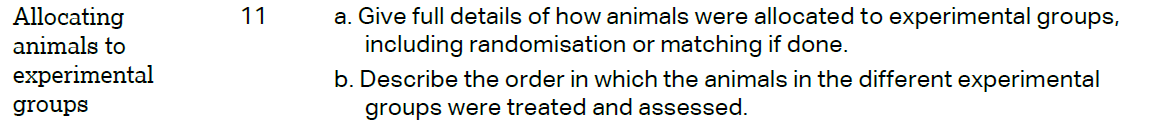 | Figure legend 4 | |
| 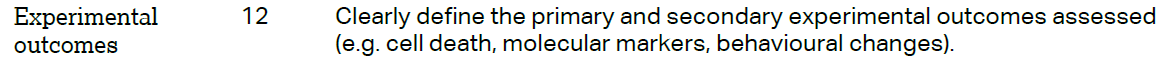 | M&M section “animal exp”paragraph 1, figure legend 4 | |
| 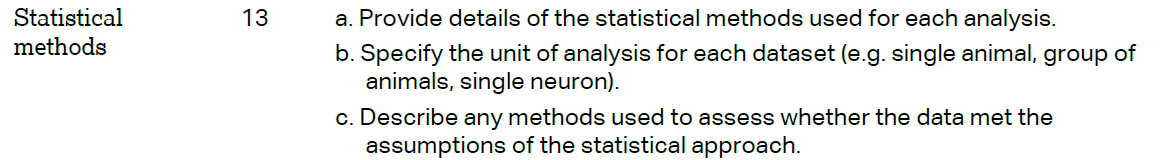 | M&M section “statistics”, figure legend 4 | |
| RESULTS |  | |
| 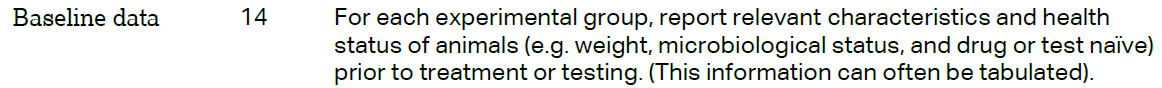 | M&M section, “animal exp.” paragraph 1 | |
| 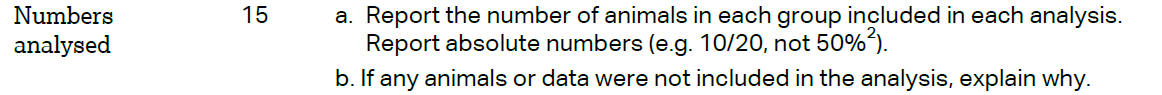 | Figure legend 4 | |
| 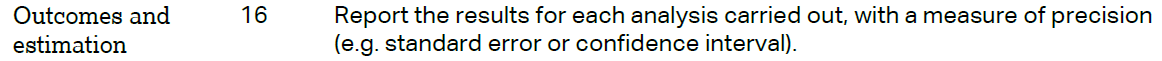 | Figure 4 | |
| 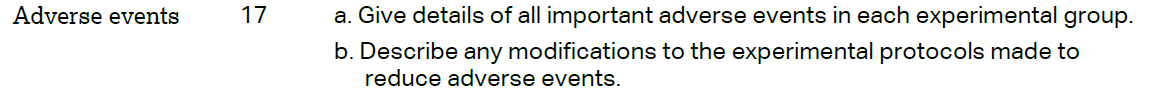 | n/a | |
| DISCUSSION |  | |
| 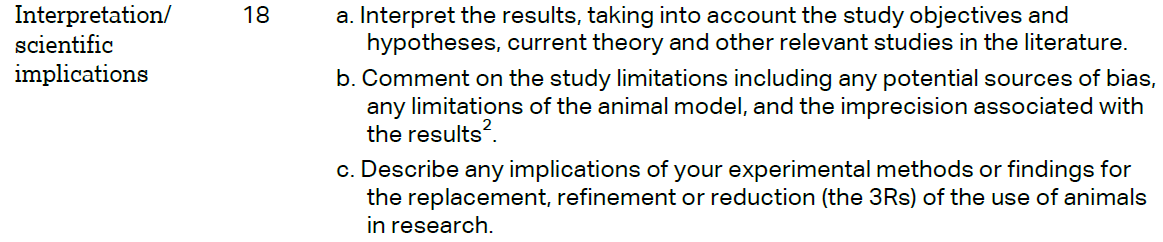 | discussion | |
| 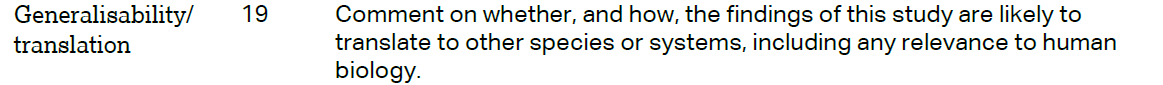 | discussion | |
| 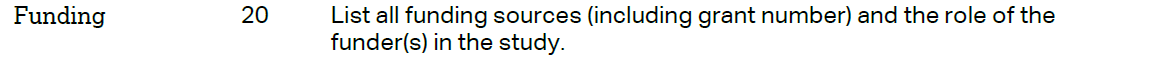 | | main body pdf-file |


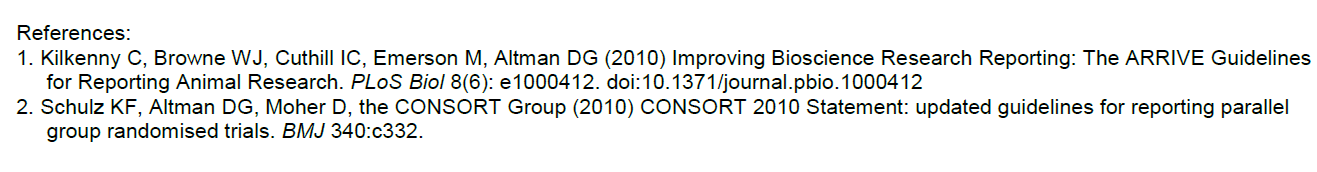

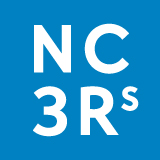

Supplement: S1 File — (DOCX) [file pone.0204942.s002.docx]
